# Failures to disagree are essential for environmental science to effectively influence policy development

**DOI:** 10.1111/ele.13984

**Published:** 2022-02-25

**Authors:** Jon Norberg, Thorsten Blenckner, Sarah E. Cornell, Owen L. Petchey, Helmut Hillebrand

**Affiliations:** ^1^ Department of Ecology, Environment and Plant Sciences Stockholm University Sweden; ^2^ Stockholm Resilience Centre Stockholm University Sweden; ^3^ Department of Evolutionary Biology and Environmental Studies University of Zürich Switzerland; ^4^ Institute for Chemistry and Biology of Marine Environments [ICBM] Carl‐von‐Ossietzky University Oldenburg Germany

**Keywords:** biodiversity‐ecosystem functioning, context‐dependent, critical transitions, locked‐in, policy making, science funding agency, scientific method, thresholds, tipping points

## Abstract

While environmental science, and ecology in particular, is working to provide better understanding to base sustainable decisions on, the way scientific understanding is developed can at times be detrimental to this cause. Locked‐in debates are often unnecessarily polarised and can compromise any common goals of the opposing camps. The present paper is inspired by a resolved debate from an unrelated field of psychology where Nobel laureate David Kahneman and Garry Klein turned what seemed to be a locked‐in debate into a constructive process for their fields. The present paper is also motivated by previous discourses regarding the role of thresholds in natural systems for management and governance, but its scope of analysis targets the scientific process within complex social‐ecological systems in general. We identified four features of environmental science that appear to predispose for locked‐in debates: (1) The strongly context‐dependent behaviour of ecological systems. (2) The dominant role of single hypothesis testing. (3) The high prominence given to theory demonstration compared investigation. (4) The effect of urgent demands to inform and steer policy. This fertile ground is further cultivated by human psychological aspects as well as the structure of funding and publication systems.

## Graphical abstract

The ecological discipline is prone to locked in debates that hamper theory development and reduce it efficiency in influencing policy development. We outline the mechanisms behind these locked in debates and discuss possible ways to prevent these.

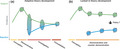

## INTRODUCTION

Doubt, debate and disagreement are central to any scientific development. Falsifying hypotheses (Popper, 1934/1959) and shifting paradigms (Kuhn, 2012) are two of many models of how theories, experiments and observations shape understanding. However, ecology also has a history of locked‐in debates, in which positions become entrenched and progress towards a consolidated consensus is hindered or even prevented by reduction in effective discourse and synthesis. Notable examples include debates on the bottom‐up versus top down controls on ecosystem organisation, the relationship between productivity and diversity, and more recently the dynamics of local biodiversity and the nature of ecosystem responses to environmental change (summarised in Box 1, 2, 3 and the following text).

BOX 1 **The diversity‐productivity relationship debate**

A splendid example of the mechanisms leading to locked‐in debate in ecology is the discussion on the productivity‐diversity relationship (PDR). Plant productivity has been proposed as an easy proxy for ecosystem functioning and biodiversity for prioritising conservation (Keddy, 2005) with significant policy implications for real world management (Huston, 1993; Tilman et al., 2006). The proposition that species richness generally follows a hump‐shaped relation with productivity (Rosenzweig & Abramsky, 1993) derives from the idea that at both extremes of a productivity gradient, species coexistence is reduced by stress and competition strength (Grime, 1973; Huston, 1979). Empirical evidence against (Adler et al., 2011) and for (Fraser et al., 2015) the ubiquitous hump‐based model has led to repetitive exchange of arguments (Fridley et al., 2012; Grace et al., 2012; Huston, 2014; Pan et al., 2012; Tredennick et al., 2016). The debate is still ongoing, and the arguments for and against the hump‐shaped relationship have been rephrased multiple times since the genesis of this theory (see the historical summary of the concept in the supplementary materia to Grace et al., 2016).

One mechanism manifest in locked‐in debates is to ***avoid the opposing view by finding reasons to exclude or dismiss it***. For example, the globally replicated study in grasslands questioning the generality of the PDR pattern (Adler et al., 2011) prompted two comments criticising the study for not using the right data. Pan et al. (2012) argued that the ‘correct’ hump‐shaped PDR would appear if the dataset were reduced to a more homogenous subset of data, whereas Fridley et al. (2012) argued that certain types of grasslands (e.g. anthropogenically managed) were under‐represented, assuming the correct pattern would emerge if the database were expanded. We are not detailing further steps in the debate here (for that, see (Grace et al., 2014, 2016; Fraser et al., 2015), but these two critiques let us point towards a second mechanism: ***avoiding engagement with the full content of the paper from ‘the other camp’***. In this case, both critiques failed to acknowledge that the original study did not at all preclude the existence of ‘humps’ in species richness at intermediate productivity but concluded on a limited predictive power and mechanistic underpinning of the PDR. Partial engagement opened for arguments that circled around technical aspects of the analyses rather than soliciting any deepened understanding of the underlying ecological interactions shaping the relationship.

BOX 2 **The dynamics of local species richness debate**

A similar example is the recent discussion about the decline in local species richness under global change. Whereas the overall decline of global biodiversity has been well documented (Díaz et al., 2019), a series of meta‐analyses has shown less straightforward consequences for the number of locally encountered species (Dornelas et al., 2014; Elahi et al., 2015; Vellend et al., 2013). These results were criticised for using a biased set of too short time series (Cardinale et al., 2018; Gonzalez et al., 2016), i.e., critiques were again focused on technical inadequacy of data, which is an easy argument to make given the complexities around reliable biodiversity data. The rebuttal to this critique was uncompromising (Vellend 2017; Vellend et al., 2017) and fuelled further argumentation that extends to recent exchanges about insect decline (Daskalova et al., 2021; Kunin, 2019; Seibold et al., 2019; van Klink et al., 2020).

This locked‐in debate reiterates the two mechanisms detailed above, as it features elaborate discussions against the evidence of the other ’camp’ and the selective rather than full engagement with the content of the opposing papers. And a third mechanism is evident in this case: ***the extension of conclusions to a related but different field***. Concluding that there is no net‐change in local species richness across monitoring time series, Vellend et al. (2013) suggested that the entire field of biodiversity‐ecosystem functioning (BEF) research was misconceived as it mainly tested for functional consequences of declining richness. This contributed to the furor in the academic exchanges, but was at the same time not really well grounded as it failed to acknowledge the reasons why richness is used so often in BEF experiments, the other aspects of diversity that have been (and are) considered, and how BEF research relates to research about increasing diversity, e.g. through the spread of exotic species.

If the different stances were fully embraced, it would become clear that there is broad agreement on the facts that biodiversity is changing locally and globally, and that human actions play a major role in this change. Instead of reporting the multidimensional nature of biodiversity (and its current changes) to policy makers, ecologists afford themselves a specious debate fuelled by the fragmentary knowledge that can be derived by measures such as richness and total biomass. This offers decision makers all options to pick strategies that might benefit other agendas, avoiding far‐reaching measures to minimise human impacts.

BOX 3 **Top‐down bottom‐up control debate**

Another locked‐in debate example with policy implications is about top‐down versus bottom‐up control on trophic relationships in ecosystems (McQueen et al., 1986). It arose from the idea that predators could limit the biomass of their prey (Hairston et al., 1960). The debate initiated a multitude of experimental studies on the role of basal resources and top predators for the organisation of food webs and ecosystems, new theories (Oksanen et al., 1981) and cross‐ecosystem synthesis efforts (Shurin et al., 2006).

While the ecological discussion was ongoing, the top‐down view was widely implemented in situations needing urgent ecosystem management. In eutrophic lakes and ponds, this took the form of biomanipulation, altering food chains with the intention of leading to more algivorous zooplankton and less phytoplankton (Carpenter & Kitchell, 1988; With & Wright, 1984). Massive human interference such as removing and killing planktivorous fish or introducing piscivorous fish or herbivores (such as mussels) resulted in short‐ to mid‐term reductions in turbidity, but the long term ecological outcomes have been less positive (Jeppesen et al., 2012). The reduction in effectiveness often comes from time‐lagged responses in the autotroph community (grazing resistance), the consumers (piscivorous fish feeding on zooplankton when young) and the ecosystem (internal re‐loading of nutrients from sediments), that is, **the discussion revolved around a oversimplified representation of a complex system**. The obvious resolution to the debate is that bottom‐up and top‐down forces interact, and that adaptive responses provided by ‘horizontal’ diversity are just as important as the vertical food‐chain interactions. The appropriate message to policy makers is that ecosystem management should not be seen as a matter for simple, direct ‘control’, i.e. there are no panaceas for ecosystem manipulations.

Detrimental consequences of locked‐in debates in ecology and in a wider sense environmental sciences reach beyond academia. Locked‐in debates reduce ecology’s impact in the shaping of environmental policies. In particular, debates in ecology often spill over into transdisciplinary development of environmental management strategies, which need to integrate multiple perspectives and stakeholders. The recent rise of science‐policy platforms and assessments such as IPCC and IPBES notwithstanding, for many ecologists the transfer of their science to management ends with formulation of advice, which ‘others’ need to transform into policy regulations and management objectives and actions. When locked‐in debates concern and reach the wider arena of society the debate can be harmful for both scientific credibility and the implementation of the understanding. Acceptance of science as a driver of policy—and acceptance of the policies themselves—increases if scientific statements are consensual (Lewandowsky et al., 2012). This motivates us to examine locked‐in debates in ecology, identify why they occur, and to find mechanisms to escape from them when they establish.

### Failures to disagree

In developing the essay that follows, we took considerable inspiration from the resolution of a locked‐in debate in psychology (Kahneman & Klein, 2009). One researcher (Klein) had spent much of his career studying executive decision making ‘in‐the‐field’ and promoting reliance on expert intuition. The other (Kahneman) had spent much of his career making experimental studies and commonly finding that intuitive judgement was flawed. The field of intuitive expertise was split into two disconnected positions: one that this expertise is real and effective, and another that it is full of flaws and biases. But by encouraging themselves to some scientific vulnerability, to lower their guards and to step into each other's shoes, Klein and Kahneman found a way out of the long‐standing debate. The result was cross‐fertilisation of two scientific perspectives, a deeper appreciation of human intuitive expertise, an improved climate within their discipline, a paper, and a friendship (Kahneman, pers. com). The paper ‘Conditions for intuitive expertise: A failure to disagree’ (Kahneman & Klein, 2009) describes how prior positions, intellectual tradition, empirical setting (e.g. field or laboratory), and standards of evidence caused a locked‐in debate. It also describes the discourse that resulted in recognition and acceptance of these differences, and hence a ‘failure to disagree’.

We believe strongly that this kind of discourse and the resulting resolution is a highly needed strategy in ecology, where environmental management decisions are often based on ideas that are still in a state of scientific emergence, yet often the need to mitigate environmental problems is urgent. The perpetuation of disagreement between ecological concepts allows for cherry‐picking a management strategy from the suite of available science concepts/understandings. The decision for a policy can then reflect other stakes (e.g. political, economic), while still justifiably claiming to be ‘following the science’. Thus, by allowing locked‐in debate to persist, ecology is undermining the role of science in policy production.

At this point we must add a critical contextualising point, and an explanation of why a group of primarily ecologists and environmental scientists engage and to some degree recast findings that have a long(er) history in philosophy of science and sociology of science. Locked‐in debates in scientific discourse are found in the study of all complex systems that are context dependent. This text primarily concerns the issue of locked‐in debates from the perspective of the involved scientists, for example, ourselves, and, we suspect, many readers of Ecology Letters. Our chosen publication venue reflects this particular introspection and we wanted to tell this story from this particular vantage point. Thus, rather than trying to mix two disciplinary perspectives in the main text, in Box 4 we connect the relevant issues we present from the viewpoint of ecologists to the core themes in the disciplines of science philosophy and sociology of science, specifically, the origin of conflicts in science, the nature of incommensurability of observations and the role of personalities, social groups and human biases.

BOX 4 **Philosophy and Sociology of Science perspectives on locked‐in debates and phases of theory development.**

Here we provide some entry points into the large and diverse fields of Philosophy of science and Sociology of Science as to how they relate to and have guided the thinking behind the specific dynamics we discuss in this paper, i.e. *locked*‐*in debates and phases of theory development*.

**FIGURE 1:**
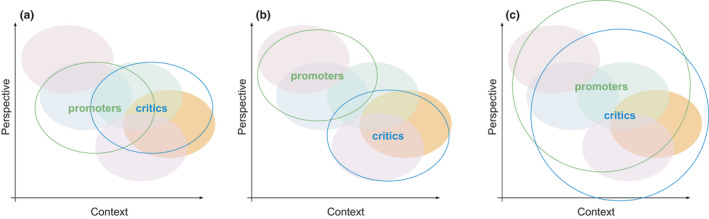
A schematic illustration of the effect of context and perspective on locked‐in debates and ways to overcome these aspects (see text for details). Black circles show the stances of promoters and critics of a given theory. Shaded coloured ovals represent different sources of evidence such as from experimental or theoretical, or different spatio‐temporal scales (perspective) or for different types of systems (context). (a) Promoters and critics of a given theory might use evidence from different contexts, such as observational vs experimental studies, and thus come to divergent conclusions that support locked‐in debate. (b) If additionally the promoters and critics come with different perspectives, their overlap becomes minimal, which solidifies the locked‐in debate. One example of different perspectives can be the scale, e.g. regional vs local scale. (c) The ability of a scientific field or group to avoid locked‐in debates and become more adaptive increases if both proponents and critics broaden their understanding of other scientific contexts and perspectives. Moreover, involving a larger diversity of research(ers) will by itself broaden context and perspective, and allow bridging and moderation among contributory evidence sources. Mediators and brokers can fill roles that link networks of different camps of contexts and perspectives

**FIGURE 2:**
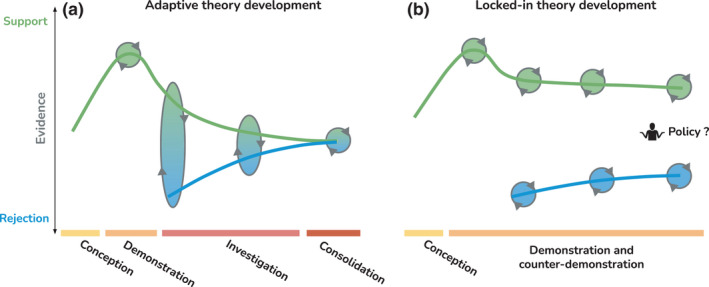
In adaptive theory development (left‐hand panel), after the initial conception and demonstration phase (the ‘Eureka phase’), demonstration studies (green) become less and investigation studies (blue) more frequent. Interaction and scientific debate leads to the reduction in variance of the reported outcomes. Consolidated evidence provides a robust basis for recommended policy. In locked‐in theory development (right‐hand panel), proponents and critics of a theory continue to publish selective cases supporting their argumentation, resulting in bimodal evidence distribution. Policy development has to choose from an unconsolidated evidence base

**Locked in debates:** First *Incommensurability*, relates to how different groups attach different meanings to an observations, data or methods through an adopted perspective (Oberheim & Hoyningen‐Huene, 2012; Sankey, 1993), originally introduced by Kuhn and Feyerabend independently around 1962 but not published until later, see (Oberheim & Hoyningen‐Huene, 2012; Sankey, 1993). Even before this, Fleck (2012) argued that different scientific perspectives and understanding come from differences in the socialisation of individuals, and the kind of implicit knowledge they bring. This provides fertile ground for conflicts that result from the different perspectives of involved stakeholders reflected in our notions of how context and perspectives differ among scientists (Figure 1). Feyerabend argued even before 1962 that experience cannot be taken for granted as an objective basis for comparing theories (Feyerabend 1957). Rather, it takes on its particular character in light of the theories we bring to it, which is why open access may relieve data from its ‘parent’ upbringing, allowing it to find a life of its own by interacting with other scientists. Second, to assume that *rational disagreement* is theoretically possible at all generally requires one to view scientists as almost superhuman, that is, being able to come to the same conclusions given the same background information and evidence (Kelp & Douven, 2012) irrespective of individual scientists’ context or historical background. But as Kuhn (1970) pointed out: ‘variability of judgement may … be essential to scientific advance’, which one can interpret as sub‐rational judgement being rather the norm and part of normal science. Whether or not one should adjust one’s stance (conciliation, Figure 2a) or remain steadfast in one’s own beliefs (locked‐in when both parties are steadfast, Figure 2b) when confronted in a peer’s opposing viewpoints depends largely on one’s appraisal of the peer's epistemic credentials, the familiarity of the evidence, the competence of evaluating the evidence and professional socialisation (Christensen & Lackey, 2013; Collins, 2010; Mulligan, 2021). This leads naturally into how scientists are biased when evaluating these credentials, as evident in analysis of the role of *social networks* in promoting or hampering scientific theories due to prominent and dominant individuals (Azoulay et al. 2019; Sun et al. 2013). Furthermore, research cliques compete with each other for attention space in journals and funding allocation by distinguishing their ideas even though these could have a common base (Bourdieu 1988; Collins 2000). This purposeful distancing can easily evolve into constructed locked‐in debates.

**Phases of theory development**: Scientific dynamics have been described by many philosophers, the most famous being Kuhn’s cycles of scientific revolution (Kuhn, 2012) including the phases of normal science, science drift, crisis and revolution and the resulting paradigm change. Graham & Dayton (2002) and Paine (2002) both question to what degree a current paradigm can be said to exist for ecology and thus, if paradigm revolutions are actually occurring in our discipline. The basic phase in Kuhn's cycle is called normal science in which observations and experiments are done in relation to contemporary understanding(s) of the system which in theory would lead to a steady evolution of ideas and understanding towards better agreement with evidence. When different camps become locked into their own understanding and interpretation of evidence one can either describe this as the precursor of science‐crisis, or alternatively, that the field is stuck in what is called pre‐science, a conglomerate of ideas and approaches with little evidential power to disprove them, waxing and waning in response to fads or ‘bandwagons’ (Paine, 2002). As discussed in the main text, environmental problems often differ from physical sciences by the diversity and adaptability of fundamental components of the study system. Ecology can thus be seen as a low‐consensus discipline sensu (Whitley, 2000) leading to a diffuse research front. This means that the finding of a counter‐example against a theory is expected in the ecological disciplines and thus model drift and crisis, the phases preceding a model revolution, is an almost permanently present aspect. This makes ecology have more of an evolutionary rather than a revolutionary dynamic (Graham & Dayton, 2002; Naeem, 2002; Paine, 2002; Tanghe et al., 2021) yet prone to lock‐in (Figure 2).

In the following we will focus on the locked‐in debate concerning the prevalence and importance of tipping points, thresholds, or critical transitions, sensu Scheffer (Scheffer, 2020). We also describe three other locked‐in debates from the field of ecology, and their negative consequences (Box 1, 2, 3). We then argue that ‘*Ecology and environmental sciences as a whole are especially vulnerable to locked*‐*in debates*’, highlighting four features that appear to predispose for locked‐in debate. Finally, we provide ‘*Pathways to escape from locked*‐*in debates* **’** by collating and synthesising existing recommendations of how to improve discourse, debate, and theory development in the environmental sciences. We amend these to address scientists, funding agencies, publishers and policy makers as parts of the policy making chain. Thereby, our perspective links to recent trends in open science and reproducibility, suggesting structural changes in values and incentives that must better align with what is good for science and policy, than with what is good for personal career development.

## THE TIPPING POINT DEBATE

The concept of tipping points seeks to detect and explain system change in terms of thresholds, critical transitions, and shifts between regimes. The idea underlying the notion of ecological tipping points was conceived and presented in the early 1990s (Holland, 1992; Holling, 1992; Scheffer et al., 1993), showing that systems with positive reinforcing and/or nonlinear interactions can have multiple attractors. When an ecosystem shifts between attractors these critical transitions lead to a new system configuration, often with beneficial or harmful consequences for people.

This theoretic understanding has had strong impacts on how systems with demonstrated feedbacks are managed (Jeppesen et al., 2012; Nyström et al., 2012; Olsson et al., 2008; Vandvik et al., 2005). The concept of thresholds and positive reinforcing feedback has also been used to suggest dimensions of planetary concern, through the planetary boundaries concept, albeit with many caveats in regard to the large scale and heterogeneity of interactions and regional dynamics of major earth system components (Steffen et al., 2015).

The theory of critical transitions creates potential for additional complexity in decision processes: due to a lack of reversibility (hysteresis), once activities result in a critical transition into an ecological state that is perceived to be harmful to society, reducing these activities does not easily restore the former state as the system may be stuck in the new attractor. Reversibility has often been an implicit assumption in environmental management at both local and global scales. Most countries have built wealth by activities that have large negative social and environmental impacts, with at least the implicit assumption that harms could be reversed if desired. Critical transitions thus increase the cost of policy errors, as being stuck in undesired attractors has to be paid for by future generations (Brock et al., 2008; Levin et al., 2013).

However, policy based on flawed assumptions of the presence of tipping points may also have negative consequences. For example, a false sense of safety with respect to gradual change might emerge as baseline shifts go unnoticed (Knowlton & Jackson, 2008; Lotze et al., 2006). Or ‐ at the other extreme ‐ the invocation of looming state shifts may lead to pessimism and inactivity regarding environmental issues (Duarte et al., 2014).

### To what degree has the debate been locked in?

Researchers active in the **development phase** of the idea of tipping points clearly stated the scientific challenge in an early paper (Scheffer et al., 2001): ‘*The notion that ecosystems may switch abruptly to a contrasting alternative stable state emerged from work on theoretical models*. *Although this provided an inspiring search image for ecologists*, *the first experimental examples that were proposed were criticised strongly*. *Indeed*, *it seemed easier to demonstrate shifts between alternative stable states in models than in the real world*’. In the following years further *demonstration* studies added to the evidence of the occurrence of tipping points in natural systems. Demonstrated jumps in time series, multimodal state variable distributions, and dual relationships with environmental drivers are some indicators of alternate stable regimes and the state dependence of driver‐response relationships (Bestelmeyer et al., 2013; Collie et al., 2004; Litzow & Hunsicker 2016; Scheffer & Carpenter, 2003). Observed abrupt shifts between clear and turbid water states in shallow lakes (Scheffer et al., 1993; Scheffer & van Nes, 2007), between dry and moist climate regimes (Claussen et al., 1999; Foley et al., 2003), in ocean and coral reef ecosystem state (Beaugrand, 2004; deYoung et al., 2008; Hare & Mantua, 2000; Mumby et al., 2013), and in a whole lake experiment (Seekell et al., 2013) are just a few apparent transitions between alternate stable states in natural ecosystems (Bestelmeyer et al., 2011; Folke et al., 2004). Knowledge of processes in these ecosystems, including demonstrations of positive feedbacks and coupled environment‐biology models, are consistent with the predicted role of feedback balance (Barkai & McQuaid, 1988; Chase, 1999; Claussen et al., 1999; Muthukrishnan et al., 2016; Scheffer et al., 1993).

During this development phase of the threshold concept, the implications for policy were becoming apparent. The irreversibility of consequences from actions was a powerful message that resonated with decision makers (Hughes et al., 2007; Lubchenco et al., 2019; Olsson et al., 2008). The theory gained much attention in scientific high profile publications as well as affecting high level policy making such as the Paris agreement and the Encyclical letter *Laudato si’* of the *Holy Father Francis* on care for our common home (Francis 2015).

The scientific community gradually moved into the **investigation phase** adding evidence questioning both the prevalence of tipping points in natural systems and the possibility to determine the position of thresholds: Observations of apparent state shifts in natural ecosystems were sometimes inconclusive (Connell & Sousa, 1983; Chavez et al., 2003; Peterson, 1984; Ratajczak et al., 2018; Scheffer et al., 2001; Schröder et al., 2005). It was noted that demonstration of a positive feedback is in itself insufficient evidence (Petraitis & Hoffman, 2010; Scheffer et al., 2001; Schröder et al., 2005). Also, the role of context such as diversity as well as spatial scale are shown to both modify and mask these phenomena (Dakos et al., 2019; Jouffray et al., 2015; van Nes & Scheffer, 2005).

Some exchanges did not seem to move towards a common understanding or identification of the cause of differences, for example: ‘*We show that notions of planetary boundaries add no insight into our understanding of the threats to biodiversity and ecosystem functioning*, *have no evidence to support them*, *are too vague for use by those who manage biodiversity*, *and promote pernicious policies*’ (Montoya et al., 2018a). *‘A recent article by Montoya* et al. *[1] in Trends in Ecology and Evolution presents a vitriolic and highly opinionated critique of the planetary boundaries (PBs) framework based on a fundamental misrepresentation of the framework and a repetition of earlier ill*‐*informed and misguided attacks on it*. *Herein we set the record straight and note more positive ways forward’* (Rockström et al., 2018). ‘*Nothing validates our concerns about Rockström* et al.*’s work more than their response to our critique of it’*. (Montoya et al., 2018b).

More recent exchanges have returned to the theoretic foundations, resulting in a much more objective tone (Hillebrand et al. 2020, 2021; Lade et al. 2021) and inviting progress towards a more investigative phase within this research field (Kéfi et al. 2019). A major synthesis effort by (Hillebrand et al. 2020), consisting of 36 meta‐analyses with 4600 environmental change studies, found little statistical evidence for threshold‐type responses along environmental pressure gradients based on field and experimental studies. They showed that this lack of evidence may result from low detectability of thresholds in empirical data, and argued that thresholds that cannot be readily detected are not well suited to defining environmental policies. They explain that these findings affect the wide range of present‐day policy narratives involving tipping points, regime shifts and planetary boundary concepts, which are based upon, at least, expectations that thresholds will be prevalent in complex systems with feedbacks and nonlinearity (Cinquin & Demongeot 2002; Kéfi et al. 2016; Marzloff et al. 2011).

This outcome of a synthesis across studies contrasts with the previously mentioned numerous single studies that report evidence of threshold type responses. Hence, at least at first sight, there appears to be evidence both for and against the conclusion that threshold‐type responses to environmental change are common and important. We believe there are many reasons for these disparate views, including the different contexts (planetary vs regional vs local scale, specific systems such as coral reefs, savannahs, temperate forests etc., field vs experimental systems) as well as the perspectives (looking for patterns vs mechanistic understanding, model driven reasoning vs experimental and field study based evidence) that are used by different scientists.

## ECOLOGY’S INHERENT VULNERABILITY TO LOCKED‐IN DEBATES

In environmental science in general, and ecology in particular, debates in which the involved parties have entrenched positions seem to be especially common and long‐lasting. Why is this? In Box 1, 2, 3, we give a few other examples of locked‐in debates that show some of the mechanisms for maintaining polarising positions. It should be mentioned that there are of course also notable examples of failures to disagree through positive interpersonal interactions within our field. One example is Boris Worm’s suggestion by extrapolation that 90% of world's fish stocks could crash by 2048 (Worm et al., 2006) which was highly criticised by Ray Hilborn, a controversy that was even debated by these proponents on US national public radio. But then, after deciding to work together within the context of an NCEAS working group they eventually found common ground resulting in a highly influential and co‐authored paper (Worm et al., 2009). In the following text, we discuss the debate regarding the prominence of tipping points in socio‐ecological systems, with a focus on (1) different perspectives on context dependent phenomena, (2) different phases of theory development, (3) urgency in policy development and (4) human biases and virtues.

### Context dependence

Locked‐in debates in ecology are fuelled by the considerable context dependence of the study systems. One team can find one result, while another could find the opposite, and both can be correct. Their stances can remain unchanged in the light of counter‐evidence, and progress towards consensus can be slow. In fact, in ecology we may never reach a narrow consensus, or a grand unifying theory, precisely because organisms and species communities adapt to complex dynamic external contexts, and thereby create unique interactions and responses. This can make the formulation of general statements and conclusions about how a system behaves and responds to environmental change difficult.

Another consequence is that in order to test a theory’s generality, studies need to be conducted under a range of conditions. In ecology, true replication is near impossible (Baker, 2016; O’Grady, 2020), except for in highly controlled lab conditions. Meta‐analysis examines if observed responses generalise across different contexts, in part by attempting to account for variation among studies caused by context (Gurevitch et al., 2018). While contributing to a posteriori generality, meta‐analysis results are, however, already constrained by researcher perspectives and biases in choice of study systems. This has led to, for example, heavy overrepresentation of results from western and rich‐world countries (Pysek et al., 2008).

Theoretical studies have a defined scope, with explicit and implicit assumptions, that can also be considered as a particular context. Additionally, researchers are susceptible to confirmation bias (Fanelli et al., 2017; Holman et al., 2015), so current theories in vogue shape the evidence that is funded, generated and published. Context dependence thus permits locked‐in debates by providing the promoters or critics of a theory or framework a certain freedom to choose different contexts to make their point (Figure 1a). Even when coming with the same conceptual perspective, they might arrive at divergent conclusions.

### Phases of theory development

Theory demonstration is a critical step in the scientific process of a newly developed theory (Figure 2). New (or extended) theories are often proposed with a certain type of empirical phenomenon in mind. For instance, the neutral theory of biodiversity (Hubbell, 2001) was derived based on observations in tropical forests (Hubbell et al., 1999). Critical transition theory (Scheffer et al., 2001) originated from observations of alternative equilibria in shallow lakes (Scheffer et al., 1993). The equilibrium theory of island biogeography was derived from observations of species‐area and species‐isolation patterns in pacific birds (MacArthur & Wilson, 1963). In the demonstration phase of theory development, considerable importance is placed on an individual study that supports (or refutes) a theory; this may be the first study, or the first in a specific context. If the theory is to have some merit beyond this initial case, it must be supported by observations in different systems and serve to explain patterns that scientists were not able to explain before. The later part of the demonstration phase consists of a search for supporting cases.

The theory investigation phase has ‘a different motivation: to evaluate the explanatory adequacy and limitations of theories so as to improve them’ (Grace et al., 2012). Whereas theory demonstration concerns the number of supporting cases (*n*), theory investigation concerns the proportion (*p*) of all *N* relevant studies that support the theory (*p* = *n*/*N*). Perhaps ideally, such investigation would consist of meta‐analyses of globally replicated studies, but as a minimum, theory investigation requires a systematic literature review to evaluate the extent of the relevant studies (*N*). Systematic reviewing has consequently developed as a toolbox with well‐defined scientific methods in recent years (Lortie, 2014; Moher et al., 2015; Hillebrand & Gurevitch, 2016). If the theory (or the prediction derived from it) allows quantitative tests, the systematic review can be extended towards a quantitative synthesis in the form of meta‐analysis to explicitly test how general the prediction is (Hillebrand & Gurevitch, 2016; Gurevitch et al., 2018).

We have the opinion that various factors lead to a preponderance of theory demonstration over theory investigation in ecological research. In our view, and acknowledging that others would prioritise different factors, various factors present obstacles to a well‐functioning theory investigation phase, including:

- Publication bias against non‐significant results, for example, (Csada et al., 1996): A case study with a clear message supporting a new theory is likely accepted by a journal, so stand‐alone examples (and counter‐examples) enter the literature despite their context‐specificity. In contrast, a paper demonstrating the limits of a theory will consist of findings that are unclear or ‘noisy’ and that are derived from earlier studies. This means it may be seen as not novel and as less important for scientific publication. Asymmetry in required scrutiny levels: studies not showing an expected pattern, with results that draw into question the validity or generality of a theory, recurrently face the criticism that the data were inapt to test the underlying theory. Demonstrating the limits of a theory often requires an exhaustive examination of the evidence, more so than when results support a theory.
- Underestimation of false positives, (Nissen et al., 2016): Researchers are often very careful in stating that absence of evidence, for example, for thresholds, is not the evidence of its absence (Hillebrand et al., 2020). However, the opposite caveat is equally true as the presence of ‘evidence’ (e.g. observing a tipping behaviour) *per se* is not yet evidence of the presence of a mechanism (e.g. threshold transgression).
- Low diversity of hypothesis, for example, (Betini et al., 2017): The simplistic all‐or‐nothing nature of hypothesis‐testing is problematic because it means that a given hypothesis likely only concerns a limited subset of causal mechanisms and contexts. This provides fertile ground for locked‐in debates because it is often tempting to inflate the generalities of a significant result for publication impact or to attract funding. But falsifying an ecological theory in one system does not necessarily require rejection of the theory as such. Given the context specificity of ecological results, even directly competing theories may be approved somewhere, thus research groups and even the discipline as such are not ‘forced’ to abandon theories. Locked‐in debates are often not so much about the scientific method itself, but researchers’ reluctance to clarify the limited contexts their findings apply to. Recognising in which context a theory successfully predicts an observable phenomenon is a major step towards adaptive theory development and maturation.

These three factors, and likely others, all lead to a situation where the development of a concept is maintained in the theory demonstration phase. In the highly contextual field of ecology, this paves the way for unproductive locked‐in debates (Figure 2, right‐hand panel).

### Urgency in policy development

Policy development sometimes outpaces the consensus findings of scientific evidence, which can contribute to the formation and persistence of a locked‐in debate. We highlight three main reasons for this (out of a potentially much more complex setting):

• *Timing* refers to the fact that policy decisions often need to be made urgently and not in a distant future after scientists have consolidated their debates. Ecology as a science is increasing in policy importance in the face of current global environmental change, so there is pressure to transmit scientific messages rapidly to research users.
• *Complexity*, from an ecologist's perspective, is a beautiful characteristic of the biosphere and potentially a causal mechanism allowing for adaptive responses to increasing cumulative human pressures. It is hard to include this complexity into management proposals, however, often leading to overly simplistic solutions for environmental problems.
• *Communication* of findings is easier and often has more impact if the message is simple. The ease of communicating clear and strong simplified positions in a debate also enhances the likelihood that these positions are turned into management guidelines. Together, this may bias scientific communications towards scientists with loud voices and simple messages.

Locked‐in debates thus often arise from simplified communication of multidimensional, nonlinear dynamics into a bivariate generalisation (Box 1, 2, 3). The top‐down versus bottom‐up debate casts trophic dynamics into a linear chain of interactions, ignoring the diversity of feeding modes with many species in intermediate roles (omnivores, mixotrophs) (Box 3). The productivity‐diversity debate collapses many complex processes onto the single axis of biomass (production) (Box 1). Simple ball and cup analogies of threshold dynamics are not able to convey the role of spatial heterogeneity, time lags, multidimensional feedbacks and adaptive responses of the involved components.

At the same time, policy‐making suffers from locked‐in debates. Without adaptive theory development (Figure 2), decision makers are left uninformed about the validity of scientific evidence and any decision can be motivated by picking the corresponding results. When stakeholders have opposing or orthogonal aims, the lack of consolidation and consensus reduces the impact of the available scientific evidence.

### Human biases and virtues

People are susceptible to a suite of limitations and biases (as well as virtues), many of which are described in the inspirational paper by Kahneman & Klein (2009). An idea, its validity and its importance, easily become intertwined with the identity and personality of a researcher. Features of our human nature and societies can create and reinforce motivation to keep believing what we currently believe, particularly if it is linked to our own perception of status and recognition. Human social dynamics make it easier to be within a dominant belief, than to oppose it, creating the mechanisms for self‐reinforcing or lock‐in (McPherson et al., 2001; Durrett & Levin, 2005). Even as individuals, we seem more likely to accept evidence that agrees with our beliefs, and less likely to accept evidence that disagrees with them (Loehle, 1987). Moreover, human social dynamics and the media are often prone to portray debates as being deeply polarised, acrimonious, and involving conflicting personalities, ignoring the middle distributions of opinions. Taking the categorical opposition is cognitively much simpler than defining one's opinion along a gradual axis (Vasconcelos, 2019) or as a position in multidimensional space, and is easier to write an engaging (though perhaps more shallow) story about.

It seems likely that these biases and fallacies interact with the previously discussed factors, as well as the mechanisms outlined in Box 1, 2, 3, to greatly multiply the risk of locked‐in debates in ecology. (Loehle 1987) pointed out that both confirmation bias (the tendency, whether conscious or unconscious, to seek evidence that confirms evidence we already have, and discount contradictory evidence) and theory tenacity (persistent belief in a theory in spite of contrary evidence) affect problem solving and proper hypothesis testing in ecology. Theory tenacity reflects the commitment to basic personal assumptions and is often linked to an emotional investment and personal involvement in ideas which is certainly an important component for the somewhat heated debates we have highlighted here.

These social and human features partly define the axis of perspective in Figure 1. If researchers come in with different perspectives (Figure 1b), the conclusions they draw from the same evidence will differ, especially if part of their perspective is also influenced by the ecological system (the context) they are most familiar with. To illustrate this, one could ask whether Steve Hubbell would have come up with the neutral theory of biodiversity (Hubbell, 2001) if he hadn't been working on tropical forests but on a system very much influenced by environmental niches such as salt‐marshes.

## TOWARDS A FAILURE TO DISAGREE

In this last section we discuss how ecology and environmental sciences more broadly can contribute to the policy decision processes while undergoing their internal dynamics, including handling conflicts and controversies. We outline these recommendations from the perspective of the scientists, giving examples of how to enable effective, efficient and accessible environmental theory development that is useful for policy. We focus our reflections on the interplay of context and perspective (Figure 1) and we base our recommendations on the steps from conception to consolidation of an ecological idea (Figure 2). We also see policymakers, funding agencies and publishers as important agents in avoiding a lock‐in, and we present their potential roles in turn (Table 1).

**TABLE 1:** Example strategies for promoting adaptive theory development and avoiding locked‐in debates during different phases and for the main stakeholders

Phase	Scientists	Publishers and editors	Funding Agencies/proposal reviewers	Policy‐makers
**General**	Avoid contributing to polarisation between groups of researchers. Foster diversity of ideas within rather than between individual researchers by taking multiple perspectives and working with multiple hypotheses	Let article categories reflect the theory development phases and make this clear in the description of article categories! Require statements of ‘stance taken’ / ‘research tradition’, and alternate ones	Allow for a broad range of project types, also those fostering generality and reproducibility. Carefully measure the amount of focus on novelty and excellence	Make sure that evidence‐based decisions mean “based on most relevant evidence” and not on dominant evidence Engage in synthesis and assessment‐based science policy platforms such as IPCC and IPBES
**Conception** The first formulation and presentation of a proposed mechanism that explains an observed pattern	Single hypothesis, resulting in yes/no answer risk leading to lock‐in debates Open approach: Start with multiple hypotheses; assess support for each. Open development of alternate hypotheses	For submitted novelty papers, strongly encourage presenting alternative ways in which the patterns may be explained. Also, require addressing the role of both external and internal (biodiversity) context dependence for the proposed mechanisms	Beware of context transferring ideas that assume that because something is true in one context it can be applied in another. Risk‐averse deliverable orientation may promote single explanation ideas, rather than multiple possibilities	Clarity about the uncertainty of a newly proposed idea/mechanism/explanation, and alternatives. Awareness of the early phase of an idea
**Demonstration** one or a few case studies in support of an idea/model	To avoid lock‐in / to promote healthy debate: Present your own stance and personal background and predisposition. Be aware of the personal motivation and perspectives for promoting or objecting to an idea. Convince by arguments and try to understand others’ arguments from **their** perspective. Revision of understanding is not a failure. Be aware of confirmation bias and homophily bias	Enforce acknowledgement and presentation of the state of the theory development process of the main idea, the sources of uncertainty, and the alternatives including evidence demonstrating the alternative mechanisms Enforce explicit statement that the demonstration does not say anything about generality, and context‐dependence is strong and common Foster open data and open code to allow re‐analysis by different researchers Publish reviews and replies	Funding large single PI projects can strongly promote idea lock‐ins! To avoid lock‐in, fund diverse approaches to the development of new ideas, not only the current dogma Foster open processes for proposal development Foster open data and open code to allow re‐analysis by different projects	Accept changes in scientific knowledge, ensure adaptive policies and management in partnership with science Identify the stances made in academia to make risk evaluations of false positives/negatives for each stance Work with academia to identify the distribution of stances and the development of these over time Decision making under uncertainty: make a cost‐benefit matrix for consequences if the theory is true OR false
**Investigation** systematic and thorough testing of an idea/model	Start with multiple hypotheses. Collaborate across stances when designing an empirical test protocol. If possible, pre‐register your hypothesis. Reach out to establish an inclusive network. Aggregate around common values and protocols while allowing these to develop When important policy implications are at stake, be aware when both “camps” actually point in the same direction of action even if they have different reasons. (I.e. they agree on what to do, but have different reasons for why that should be done)	Have targeted article categories for theory investigation. These are not only meta‐reviews but also allow articles with either/both non‐significant results and repeating studies. Investigate methods to amplify researcher credits of such less citation‐attracting articles	Make investigation contracts with different success valuation criteria compared to the development of new ideas Promote international networks that investigate important ideas in different contexts. Explicitly fund projects that reproduce science or include synthesis aspects	Focus on decision‐making under context‐dependence and uncertainty: avoid panacea solutions In co‐designed projects, request systematic review and other evidence‐based synthesis efforts
**Consolidation** A broader agreement on the supported conclusions and their uncertainties	Detailed analysis, many systems, reviews and special cases are important. Engage in synthesis and modularised studies spanning multiple contexts If you are a brokering/bridging personality, initiate open and inclusive consolidation projects	Require or invite consolidation articles that are produced by a group of researchers spanning the range of stances, perspectives and conclusions	Support stakeholder integration tools such as decision support systems. Continued support of the investigation phase. Credit non‐novel results. The creation and maintenance of synthesis centres such as NCEAS, SESYNC, and iDiv provide one formal way to bring diverging opinions together. Perhaps even targeted invitations to opposing views and people able to build bridges from topics that are becoming locked in by centre leaders is an option	Identify organisations and groups that are credible to make the consolidation in collaboration with academia Require science funding agencies to provide means for establishing the state of the art consolidation reports on policy‐relevant issues Base decision on broad assessments from science

We, as the author team of this article, also consider ourselves to be on an intellectual journey from understanding the consequences of implicit and explicit biases to digesting and implementing recommendations for a better discourse in the environmental sciences. Thus, we do not want to prescribe certain actions, but offer our current perspective which partly reflects our own learning trajectory while discussing these issues and partly are a promise to our future selves.

### Among scientists

If scientists ‐ individually as well as a group ‐ allow themselves to broaden the contexts and perspectives of their work (Figure 1), we are confident that chances for a failure to disagree will increase. A few recommendations towards this goal are in general, and some specifically inspired from the tipping point debate:

*Address the same question*: Before locking‐horns regarding different results, carefully examine the precise questions being addressed by different studies, and ensure they are similar enough for meaningful and productive comparison and debate. If questions are different don’t take for granted that your question is necessarily the most important one.

*Adopt, promote, and expand open science*: Much of the concise recommendations for scientists are derived from ongoing discussions about open and reproducible science (Fraser et al., 2018; Powers & Hampton, 2019).

*Actively take multiple perspectives*: This includes collaborating with, and understanding the perspective of, a diverse group of colleagues, especially when camps have already been built. It often requires persons outside these camps to bring scientists with very opposing views together to produce a synergy perspective. To be fruitful, the coordinator of such a consolidation has to be accepted as an honest broker, but more importantly it requires the willingness to lower the guard and take on the extra effort to interact with colleagues challenging one's own perspective. Also to reach out across the divides and suggest writing a paper that together will define the different stances, their arguments and their main disagreements rather than write papers that fortify the single perspectives. Here it is important to come to this endeavour with a mindset of respect for the other's perspective and trying to understand what contexts make them come to another conclusion, not to prove your own.

*Pre‐register studies and hypotheses*: The demonstration and investigation phases are especially prone to confirmation biases. Pre‐registration would undermine undesirable temptations to develop hypotheses after the results are known and will reduce publication bias towards significant results. Registries exist (https://www.cos.io/initiatives/prereg, https://aspredicted.org/) and are part of open science strategies. Part of this is the commitment to FAIR data principles (Wilkinson et al., 2016). It can hardly be overestimated how much open data policies have revolutionised the scientific landscape over the last few decades as data are no longer entries in lab books and on local hard drives, but are shared with publications and covered by their own digital object identifiers.

*Promote re‐analysis of scientific questions*: Open science tools and open data are also prerequisites for consolidation, because they allow and facilitate re‐analysis of scientific questions from a different viewpoint and merging of multiple data sources into quantitative synthesis. The rise of meta‐analyses in the environmental sciences (Gurevitch et al., 2018) allows the generation of central tendencies across a wide range of (context‐specific) case studies (appropriate quality criteria are essential for conducting a review synthesis and meta‐analysis (Koricheva & Gurevitch, 2014; Nakagawa et al., 2017)).

*Employ multiple‐hypothesis approaches*: To avoid falling in the trap of looking for a particular causal explanation, researchers studying highly context dependent complex systems such as ecological ones might want to refrain from a single hypothesis falsification method and employ multiple‐hypothesis approaches focussed on establishing estimates of different explanatory models likelihoods (Betini et al., 2017; Chamberlin, 1890, 1965). These can be seen as putting up a set of scenarios of how the study system might work, and through iteration and reformulating them work towards a better understanding (Brittan & Bandyopadhyay, 2019). Platt (1964) suggested a strong inference by testing multiple hypotheses across multiple experiments and evaluating how evidence for each varies with context (similar to in meta‐analyses, though with multiple hypotheses. Approaches such as causal analysis (Larsen et al., 2019; Laughlin & Grace, 2019) and hierarchy‐of‐hypotheses (Heger et al., 2021) are promising ideas to further enhance the theory building in ecology and beyond.

*Understand and protect against our cognitive biases*: Betini et al. (2017) argue for multiple hypotheses as besides confirmation bias also pattern seeking (human are known in finding patterns even where there are none to be found) and belief bias (when the data are wrong, but tell us something that we are expecting, we tend be less rigorous in our assessment of the evidence). These three cognitive biases should serve as a reminder to formulate multiple working hypotheses (more than two) and is at the same time an explanation why scientists are failing to do so (Betini et al. 2017). In practice, if authors who propose a new mechanism, model or theory can take as a habit to also include alternative hypotheses that could generate similar patterns, rather than trying to argue for watertight proofs, the sense of vulnerability when the main idea gets challenged may be emotionally not as sensitive as when one’s full reputation feels at stake. Making their own stance explicit in formulating an idea also helps other scientists to understand on which fundament a new concept has been developed.

*Perform modularised studies*: These offer a different path for scientists to engage in multi‐perspective research. The replication of observations and experiments in different locations offers an unprecedented statistical power to overcome context‐specific results (Borer et al., 2014; Duffy et al., 2015; Kemppinen et al., 2021). Additionally, already the discussion towards such an endeavour contributes to avoiding lock‐in debates as it requires to integrate different views on which question to pose and prioritise and how to analyse the results. Not all environmental research should be ‘general’ and synthesis oriented ‐ by contrast: the best information to address a system‐specific question can be detailed and system‐specific. Bringing local data into a broader context reduces the bias imposed by a single perspective. Environmental sciences could even go a step beyond and select issues that are of international importance and have international theory investigation contracts.

*Change assessment criteria and incentive structures*: Hiring processes that focus on the impact factor of journals in which applicants have published are being replaced by processes that consider more holistic criteria (e.g. as is being encouraged by DORA), including incorporation of open science activities (e.g. HI‐FRAME).

### Interacting with funding agencies and publishers

Funding agencies and publishers are two main facilitators of scientific knowledge production as they enable the research to be done and publish the results to an increasingly diverse audience. Therefore, they can act as important multipliers by requesting open data and open science compliance. Given their role as alpha and omega in this scientific process, they can enforce pre‐registration of studies, and publication of FAIR (findability, accessibility, interoperability and reusability, (Wilkinson et al., 2016)) data and code as described in the section above. Whereas data publication often already is mandatory for receiving funds and publishing manuscripts, it often does not have a quality assessment that analyses whether data are actually FAIR (Roche et al., 2015), which often limits assessments of the reproducibility of the study.

Additional advice we can only share from our role as scientists, even though all of us are working in reviewer and editor roles for funding bodies and publication outlets. From this perspective, we see potential impacts in addition to these gatekeeper and multiplier functions. These either promote a larger diversity of perspectives or enable research in multiple contexts (Figure 1).

At the funding agency level, recent years have seen an increasing tendency towards hypercompetition that uses narratives of ‘excellence’ to focus large proportions of funding on single persons or centres (Moore et al., 2017). Such centralising funding schemes pave the way to lock‐in debates, as they foster the predominance of single perspectives to the disadvantage of diversity in stances, approaches and ideas. In addition to researcher excellence, novelty and feasibility of a project are two further traits of successful proposals. Although novelty should foster risk‐taking and feasibility risk‐aversing, both can contribute to locked‐in debates. The novelty argument often prevents redoing a study in a different setting, for different organisms, and at different temporal and spatial scales. The feasibility argument fosters focusing on single testable hypotheses rather than a multiverse of contrasting hypotheses. Thus, funding agencies can actively contribute to avoiding locked‐in debates by supporting diverse assemblages of researchers, enable networking, enforce open science formats, and explicitly provide funds to redo studies.

Publishers and editors also play a key role to *enforce pre*‐*registration of studies*, *and publication of FAIR data and code* and to *change assessment criteria and incentive structures*. Furthermore, they can provide explicit outlets for ideas and criticism to existing ideas, as can be exemplified by the Forum section in *Oikos* and Ideas & Perspectives at *Ecology Letters*. When publishers and editors *openly enforce and mediate a process of effective discourse* that is itself published, this may prevent seemingly unproductive series of critiques and rebuttals that sometimes appear in the literature.

Labelling manuscripts in the framework of conceptualisation, demonstration, investigation and consolidation steps would help readers understand the motivation of research. For submitted novelty papers, there should be strong encouragement to present alternative ways in which the patterns may be explained. Additionally, fostering theory investigation over demonstration, targeted formats for meta‐studies and reproduction of existing studies.

### Interacting with policy‐makers

As pointed out by Arrow & Fisher (1974): ‘Any discussion of public policy in the face of uncertainty must come to grips with the problem of determining an appropriate attitude toward risk on the part of the policy maker’ and that ‘the expected benefits of an irreversible decision should be adjusted to reflect the loss of options it entails’. Irreversibility thus induces an added complexity to decision making (Levin et al., 2013) requiring a reduction of the expected benefits which are then balanced against costs (Arrow & Fisher, 1974).

The scientific process is integral to society's capacity to estimate these uncertainties in risk (both probabilities and impacts). Theory demonstration raises the awareness that implicates the possibility of thresholds which fundamentally changes the decision problem (Arrow & Fisher, 1974). The investigation phase reduces uncertainty in position and likelihood of threshold for a particular system. The fact that environmental systems, and in particular ecological processes, are complex makes them extra prone to the mechanisms that foster locked‐in debates, reducing the effectiveness of the environmental discipline to affect policy towards better informed decisions.

It rests on all stakeholders of scientific theory development to be aware of the dynamics of the social mechanisms that can reduce the capacity of the science scholar system as a whole to efficiently move towards better match between proposed models of explanation and real world dynamics (Biggs et al., 2009; Brittan & Bandyopadhyay, 2019).

### Accounting for scientific uncertainty in the policy context: the tipping point example

**FIGURE 3:**
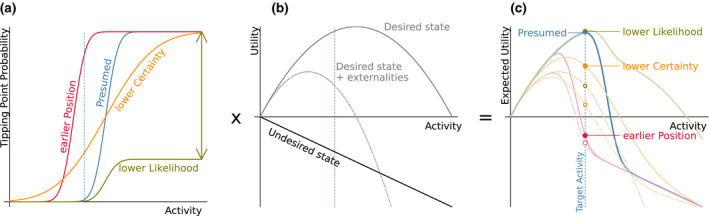
A conceptual illustration of part of the science‐policy interface from the perspective of tipping points. Expected utility (c) is the sum of the utility of each state (b) times the probability of that state as a function of the (human) activity (a). While this expected utility could be a direct driver of policy, in practice it is likely to influence policy alongside, or even subordinately to other factors. **Panel a**: Scientific inquiry is trying to establish where the position of a tipping point is, the degree of confidence in the estimate of the position, and the likelihood of a particular system to be prone to flip as one increases the level of some human activity. **Panel b**: The expected utility that society can derive from either the desired or the undesired states are also subject to scientific estimates. The cost of unaccounted externalities (dashed line) depends on both the scientific process, as well as the willingness of decision‐makers to incorporate all stakeholders’ costs. **Panel c**: The resulting potential outcomes (probabilities times utilities of the different states) are shown in relation to the scenarios presumed by science to the decision‐makers (blue line). The coloured filled circles represent the consequence of scientific misjudgement, that is, if the position was in fact earlier (red), the certainty of the position lower (orange) or the likelihood of a tipping point occurring lower (green) given a target activity that is based on the presumed scenario (blue). The consequence of unaccounted externalities is also shown (corresponding dashed lines and open circles). The vertical lines highlight the difference in expected utility between the scenarios if the management policy at the optimal utility for the presumed scenario is chosen. Note that if the management decisions surpass the optimal activity of the presumed scenario (top of blue line), most alternative scenarios will show a very rapid decline in total utility

Science's role in decision making is often fundamental, but science is neither infallible nor fast to reach a consensus. Hence there is a need to account for the consequences of mistakes and uncertainty, that is, to account for the consequences of the scientific process in the context of decision making. Here, we consider the social cost of under‐ or overestimating the prevalence of and uncertainty around potential tipping points. Inspired by (Dasgupta, 2021; Lenton et al., 2019), we demonstrate risk assessment in finding an estimate of the potential position, likelihood and uncertainty of a threshold for a given activity, and the uncertainties and scope of potential outcomes (Figure 3) thereof. Scientific uncertainty enters first in estimates of the position, certainty and likelihood of tipping point positions, which together determine the shape of the relation of the probability for a tipping point with increasing human activity (Figure 3a). Science also contributes to the estimates of utilities gained in each state (Figure 3b). These estimates of utility can include indirect consequences of human activity and externalities. An example of the latter could be when the management of the agricultural sector is based on expected agricultural output (solid gray line) but the negative outcomes in terms of biodiversity loss and eutrophication are not internalised in this decision process (gray dashed line). The solid black line represents an undesired state where feedback processes keep the system in a state of increasing costs with activity.

During the scientific development process, understanding is by definition incomplete, hence scientific misjudgements in the position, certainty and likelihood of a tipping point will be likely. The potential consequences of scientific uncertainty and misjudgement can be investigated based on the optimal expected utility of the presumed scenario (Figure 3c).

The main difficulty for tipping point prone systems is when the presumed but misjudged optimal target level of activity, in reality, leads to a high risk of a critical transition into an undesired attractor (Figure 3c, red lines and circles), akin to believing and acting as if a cliff‐edge is further away than it really is, and so risk falling off the cliff. In the science‐policy interface, scientists therefore call for precautionary measures by pointing at the possibility of a critical transition and for the dire consequences of the undesired state. If, however, the scientific estimate of the position of a threshold is too precautionary, revenues from higher levels of activity are lost opportunity costs.

Another danger lies in being too confident (in relation to real uncertainty) about the threshold position. If the blue line illustrates the presumed certainty, but in reality the probabilities for a particular system to flip are much more variable (orange lines), this false certainty moves the target level of activity into a region where the probability of a tipping point to occur increases rapidly and would incur a loss of utility.

If the likelihood of a tipping point is much lower than presumed (green lines and circles), the lost opportunity cost is negligible for the utility scenarios (panel b) we have chosen for this illustration, that is, the cost of the precautionary approach is low. Costs may increase from an inadequate use of thresholds in management, when thresholds (i) become ‘targets’, such that activities increase more than they would have in the absence of recognising this threshold or (ii) become inflexible static instruments in a dynamic setting (given the time needed to change once implemented).

If the expected utility of the desired state is largely overestimated, for example, if its negative externalities are unaccounted for (dotted lines), the target level of activity may be set too high. An example of this is the use of fertiliser in agriculture to promote agricultural revenue, but not accounting for the impact of eutrophication such as toxic blooms and reduced utility for coastal recreation which is a much more distributed cost compared to the revenue from agriculture and therefore less easily accounted for. We find that proportionally, this effect is largest for the green scenario where the risk of tipping points is, in reality, lower than presumed (distance between filled and open green circles). There is also a risk that too much focus on tipping points can make scientists and policy makers pay less attention to externalities that can be potentially more severe than the (moderate in the green scenario) risk of crossing a tipping point.

Even if scientific debates are present regarding the position, uncertainty and likelihood, in the overwhelming number of contexts the consequence for management seems to be similar, to err on the precautionary side is less costly than to squeeze out the maximum outcome based on uncertain understanding. The general pattern of the different scenarios of course depends on the parametrisation of the utility curves in relation to the probability curves and we have chosen the presented scenarios for illustration purposes (notebook is available for trying different scenarios). With the given parametrisation, precautionary thinking has far higher expected positive outcomes than trying to find the optimal level of activity based on uncertain understanding (Hassler et al. 2018).

The impact of science is, perhaps unfortunately, linked to how clear and consolidated a message is provided. At times, scientific discourses obfuscate the fact that even opposing camps in the scientific community that argue for different perspectives may in the end deduct the same policy recommendation, for example to reduce fossil fuel emissions or preserving biodiversity even though the reasons for their conclusions may differ largely. While disagreements are needed in the investigation process of developing understanding, a *failure to disagree* is needed if the suggested management implications are comparable or even the same from different (potentially disagreeing) camps of the scientific community.

## CONCLUSIONS

We have tried to highlight some of the processes of scientific discourse that lead to reduced effectiveness of environmental science to provide much‐needed management and governance advice. Scientific debates, controversies and even conflicts are needed, useful and even fun. But locked‐in debates can not only stall scientific progress but also significantly reduce the usefulness of science for societal decision making. We have found it useful to be aware of three main aspects that may change the state of mind in how we interact amongst colleagues in the environmental sciences: (1) Being aware of the different perspectives used, and contexts that researchers work in, (2) understanding the sequence of theory development and one's own role in it, and (3) being aware of the policy consequences of unavoidable uncertainty in scientific predictions and estimates.

Humanity is at a crossroads and we cannot afford weak science, nor touting solutions based on evidence that is selectively chosen. Human decision‐making capacity will benefit if science‐policy interfaces, as well as publishers, funding agencies and policymakers are able to transparently reduce human biases and personal gains from the effort of improving our common understanding. Most importantly, we suggest that reaching out to opposing camps to reach a common and diverse understanding of the questions, contexts and perspective, rather than fortifying one's own view, is at the heart of unlocking debates. Failing to disagree, as happened for Kahneman and Klein, not only creates a more effective and robust scientific process but also leads to friendships.

## AUTHORSHIP

All authors contributed to ideas and writing of the manuscript.

### PEER REVIEW

The peer review history for this article is available at https://publons.com/publon/10.1111/ele.13984.

## Data Availability

No data are used in this manuscript.
